# Comparison of Ondansetron versus Domperidone for treating vomiting in acute gastroenteritis in children at a resource limited setting of South Punjab, Pakistan

**DOI:** 10.12669/pjms.38.6.5532

**Published:** 2022

**Authors:** Tauseef Ahmad, Uzma Zarafshan, Bushra Sahar

**Affiliations:** 1 Tauseef Ahmad,Department of Pediatrics, Tehsil Headquarter Hospital, Liaqat Pur, Pakistan; 2 Uzma Zarafshan,Department of Obstetrics & Gynecology, Tehsil Headquarter Hospital, Liaqat Pur, Pakistan; 3 Bushra Sahar, Department of Obstetrics & Gynecology, Tehsil Headquarter Hospital, Liaqat Pur, Pakistan

**Keywords:** Acute gastroenteritis, Domperidone, Ondansetron, dehydration

## Abstract

**Objectives:**

To compare the efficacy of Ondansetron versus Domperidone for treating vomiting in acute gastroenteritis (AGE) in children at a resource limited emergency setting of South Punjab, Pakistan.

**Methods:**

This open label randomized controlled trial was conducted at The Pediatric Emergency Department of Tehshil Headquarter Hospital, Liaqatpur, Pakistan, from July 2020 to June 2021. A total of 300 children of both genders aged below 12 years of age having 3 or more non-bilious, non-bloody vomiting episodes within 24 hours and with suggestive signs and symptoms of AGE were enrolled and randomized (150 in each group). Efficacy of both drugs was compared in terms of need of 2^nd^ dose within 15 minutes, cessation of vomiting at 6-hour and 24-hour follow up.

**Results:**

Out of a total of 300 children, 162 (54.0%) were male. Mean age was 4.7±2.3 years. Twenty seven (18.0%) children in Ondansetron group required 2^nd^ dose within 15 minutes while 38 (25.3%) children in Domperidone group required the 2^nd^ dose (p=0.1232). Cessation of vomiting at 6-hour interval was noted among 126 (84.0%) children in Ondansetron group in comparison to 118 (78.7%) in Domperidone group (p=0.2359). It was revealed that 127/142 (89.4%) children in Ondansetron group had cessation of vomiting at 24-hours follow up while this was noted to be among 108/134 (80.6%) children in Domperidone group (p=0.0390).

**Conclusion:**

In comparison to Domperidone, Ondansetron was found to have better efficacy aiming cessation of AGE associated vomiting among children with mild to moderate dehydration.

***Clinical Trial Registration:*** NCT05076461 (www.clinicaltrials.gov)

## INTRODUCTION

Acute gastroenteritis (AGE) is considered to be an important cause of acute vomiting in paediatric population.^1^ Pediatric AGE is known to be one of the commonest reasons for visits to paediatric emergency department (ED). Data from United States of America showed that around 1.5 million children below 5 years of age are diagnosed with AGE every year while AGE is also responsible for 13% of all hospitalizations.^2^ Studies from Pakistan,^3^ India^4^ and Nepal^5^ have revealed prevalence of AGE among children to be 29 to 37%, 22% and 36% respectively.

The most commonly occurring complication of AGE is dehydration while vomiting is known to be the most annoying clinical presentation. Vomiting is a cause of worry and distress, not only for the child but also for the caregiver and can cause dehydration which might need hospitalization for emergency care.^6^ Oral rehydration therapy (ORT) is the cornerstone of treatment among cases of mild to moderate dehydration because of AGE. Global societies have not yet fully endorsed antiemetic drugs as part of the pharmacological treatment options as diarrhea is one of the known side effects of these drugs.^7^

Variety of antiemetic drugs like promethazine, prochlorperazine or metoclopramide are available and frequently used off-label for the prevention and reduction of vomiting in pediatric population with AGE. Domperidone, a dopamine receptor antagonist is a frequent choice for treating vomiting in pediatric population with AGE. Ondansetron, a serotonin antagonist is also considered a useful option and thought to have fewer side effects as compared to its contemporaries.^8^ A local study from Karachi evaluating effectiveness of Ondansetron versus Domperidone aiming cessation of vomiting among children below five years of age revealed that at 24 hours, 95% of children with Ondansetron administration had cessation of vomiting in comparison to 85% with Domperidone (p=0.01).^9^ Most of the trials comparing Ondansetron and Domperidone have been conducted among children aged below five or six years of age while no study from Pakistan has compared efficacy of these antiemetic agents among children up to 12 years of age.

This study was aimed to compare the efficacy of Ondansetron versus Domperidone for treating vomiting in acute gastroenteritis in children at a resource limited emergency setting of South Punjab, Pakistan. The findings of present study were thought to give valuable evidence about the effectiveness of these antiemetic agents to prevent vomiting among children suffering with AGE at a resource limited setting of South Punjab, Pakistan.

## METHODS

This open label randomized controlled trial was conducted at The Paediatric Emergency Department of Tehsil Headquarter Hospital, Liaqatpur, Pakistan from July 2020 to June 2021. As no formal Ethical Review Committee existed at the study place, approval was acquired from the Incharge (Medical Superintendent) of the Institute (Ref No.: MS/1974, Dated: 28-08-2021). This trial was also registered at www.clinicaltrials.gov as “NCT05076461”.

Informed written consent was sought from parents/guardians of all study participants. A sample of 284 (142 in each group) considering 2-sided confidence level as 95%, power 80%, ratio of cases to control as 1, proportion of children with cessation of vomiting with Domperidone as 85% and cessation of vomiting with Ondansetron as 95%.^9^

Inclusion criteria was children of both genders aged below 12 years of age having three or more non-bilious, non-bloody vomiting episodes within 24 hours and with suggestive signs and symptoms of AGE. Sign and symptoms of AGE consisted of diarrhea with or without fever, abdominal pain and bloating/discomfort. Children who took any kinds of antiemetic in the last six hours of presentation of emergency department were excluded. Children having chronic liver disease, chronic kidney disease or congenital heart disease, neurological disorders, any kinds of malignancy, severe dehydration (requiring intravenous fluid replacement), severe acute malnutrition (weight-for-height below -3 standard deviation (SD) adopting WHO child growth protocols) or history known to allergy to antiemetics were also not enrolled.

A total of 300 (150 in each group) children fulfilling the inclusion and exclusion criteria were included and randomized through lottery method for this study. Children in Ondansetron group received oral suspension of Ondansetron as 0.15mg per kg body weight while in Domperidone group, oral suspension of Domperidone was given as 0.5 mg per kg body weight.^10^ Children in both study groups were administered designated drugs in the emergency department. In case a child had a vomiting episode within 15 minutes after giving the drug, 2^nd^ dose of the same drug (Domperidone or Ondansetron as per grouping) were given within 15 minutes after the 1^st^ dose. After 30 minutes of the administration of the study drugs, children were asked to intake fluid orally. The ORT was allowed if a child was vomiting free after 45 minutes of administration of the study drugs. Children were observed in the emergency department for 6-hours and discharged if they were vomiting free. At home, parents/guardians were asked to repeat the designated antiemetic in the same dose as given earlier if an episode of nausea or vomiting occurred but not before a gap of eight hours from the last dose. All children were asked to follow up after 24 hours for the assessment of the effectiveness of the treatment for cessation of vomiting. If a child had vomiting episodes after six hours of initial administration of study drugs, reassessment about the hydration status of the child was done and decision about admitting in the pediatric unit was made. Children who turned out to have severe dehydration after 6 hours of initial treatment were excluded from the study and admitted and managed in the paediatric unit. At 24-hour follow up, parents were asked about any episodes of vomiting during the home stay. In case, no episodes of vomiting were noted during children’s stay at home, antiemetic drugs were stopped and patients were continued on ORT. At 24-hours follow up in both study groups, efficacy was labeled in terms of number of children reporting with no episodes of vomiting for the last 24 hours. All the study information was recorded on a pre-designed proforma made specifically for this research.

Analysis of the study data was made employing SPSS version 26.0. Frequency and percentages were calculated for qualitative variables while mean and SD were calculated for quantitative data. Qualitative study variables along with efficacy were compared between both study groups utilizing chi-square test whereas independent sample t-test was used to compared quantitative variable data. P value<0.05 was labeled as significant.

**Fig.1:**
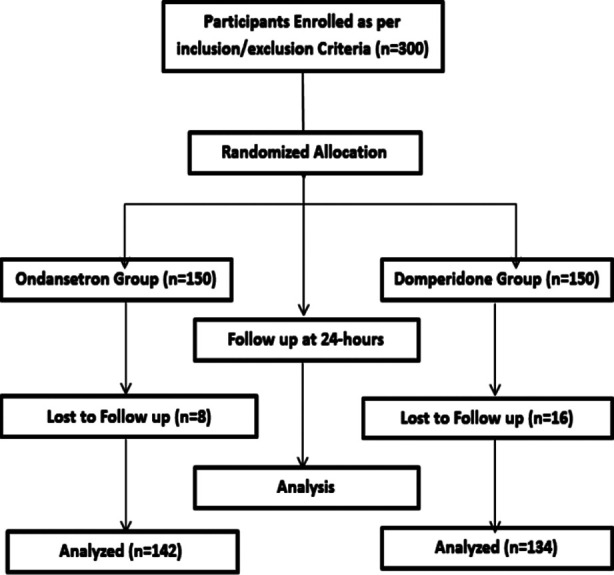
Methodology Flow Chart.

## RESULTS

Out of a total of 300 children, 162 (54.0%) were male. Mean age was calculated to be 4.7±2.3 years. Overall, mean vomiting and diarrhea episodes were noted to 7.1±3.3 and 6.2±2.9 respectively. Mild to moderate dehydration was recorded among 211 (70.3%) children. Maternal education status of 85 (28.3%) children was illiterate Table-I is showing comparison of baseline characteristics of children in both study groups and no statistically significant difference was found (p>0.05).

**Table I:** Comparison of Baseline Characteristics in Both Study Groups (N=100).

Characteristics		Ondansetron Group (n=150)	Domperidone Group (n=150)	P-Value
Gender^	Male	84 (56.0%)	78 (52.0%)	0.4870
	Female	66 (44.0%)	72 (48.0%)	
Age in years*		4.6±2.2	4.9±2.5	0.2708
Vomiting Episodes in the last 24 hours*		6.9±3.6	7.3±3.0	0.2967
Diarrheal Episodes in the last 24 hours*		6.0±2.8	6.4±3.1	0.2418
Dehydration (Mild to Moderate) ^		102 (68.0%)	109 (72.7%)	0.3763
Maternal Educational Status as Illiterate^		38 (25.3%)	47 (31.3%)	0.2489

^ Frequency and percentages calculated and chi-squared test applied for comparison.

* Mean and standard deviation calculated and independent sample t-test used for comparison.

Comparison of outcomes between children of both study groups during emergency department stay is shown in Table-II,. It was noted that 27 (18.0%) children in Ondansetron group required 2^nd^ dose within 15 minutes following 1^st^ dose while 38 (25.3%) children in Domperidone group required the 2^nd^ dose (p=0.1232). Cessation of vomiting at 6-hour interval was noted among 126 (84.0%) children in Ondansetron group in comparison to 118 (78.7%) in Domperidone group and no statistically significant difference was found (p=0.2359).

**Table II:** Comparison of Outcomes between Both Study Groups during Emergency Department Stay.

Outcomes	Ondansetron Group (n=150)	Domperidone Group (n=150)	P-Value
Second Dose Required within 15 minutes after 1^st^ Dose	27 (18.0%)	38 (25.3%)	0.1232
Cessation of Vomiting at 6-hours	126 (84.0%)	118 (78.7%)	0.2359

A total of 176 children came back for 24 hours follow up while 24 children lost the follow up so were excluded in the final analysis about the comparison of efficacy between the both study groups in terms of vomiting cessation. It was revealed that 127 (89.4%) children in Ondansetron group had cessation of vomiting at 24-hours follow up while this was noted to be among 108 (80.6%) children in Domperidone group while the difference between both study groups was found to be statistically significant (p=0.0390). No major side effects of the studied drugs were noted in any of the children.

## DISCUSSION

In the present study, 18.0% children in Ondansetron group required 2^nd^ dose within 15 minutes following 1^st^ dose while 25.3% children in Domperidone group required the 2^nd^ dose and the difference between study groups was not statistically significant (p=0.1232). Marchetti F et al. evaluating effectiveness of Domperidone versus Ondansetron among children with AGE revealed that 6.7% children in Ondansetron group and 18.5% in Domperidone group required 2^nd^ dose because of vomiting within 15 minutes following the 1^st^ done. The same study also found that 11.8% children in Ondansetron group versus 25.2% in Domperidone group required nasogastric or intravenous rehydration (p=0.008).^10^ Several studies have concluded that Ondansetron results in significant reduction in number of subjects needing intravenous therapy.^11^,^12^

We noted that cessation of vomiting at 6-hour interval was noted among 84.0% children in Ondansetron group in comparison to 78.7% in Domperidone group (p=0.2359). A local study from Karachi evaluating efficacy of Domperidone versus Ondansetron among children below five years of age with AGE shared that 13.1% children Ondansetron group had persistence of vomiting at six hours following treatment while 18.8% in Domperidone group had persistence of vomiting (p=0.21).^9^

In this study, Ondansetron proved to be significantly more effective versus Domperidone aiming cessation of vomiting at 24-hours follow up (89.4% versus 80.6%, p=0.0390). A study from Thailand by Rerksuppaphol S et al analyzing efficacy of Domperidone (at a dose of 2.5mg for children weighing below 15kg, 5mg for 15-30kg and 10 for more than 30kg) versus Ondansetron (2mg for children weighing below 15kg, 4mg for 15-30kg and 8mg for more than 30kg) among 73 children up to 15 years of age with AGE found that cessation of vomiting was noted 62% children in Ondansetron group and 44% in Domperidone group (p=0.16).^13^ As the sample size was relatively small in Rerksuppaphol S et al. trial, it was difficult to endorse the outcomes found in that study.^13^ A local study from Karachi noted that 4.9% children in Ondansetron group versus 14.5% in Domperidone group had persistence of vomiting at 24 hours after initiation of treatment (p=0.01) which is quite close to what we noted.^9^ A trial from Italy by Marchetti et al. concluded that Domperidone was not effective for treating symptomatic vomiting among children with AGE.^10^ The findings of this study are aligned with a systemic review evaluating use of Ondansetron among children with AGE where the authors proposed that guidelines about the treatment of pediatric AGE should be revised and include use of oral Ondansetron in case vomiting occurs after 1^st^ dose of ORT.^11^ Different in findings in terms of effectiveness of Domperidone versus Ondansetron could be due to difference in inclusion criteria and variation in dosage strategies of these drugs among different researchers.

As our study noted Ondansetron to have better efficacy than Domperidone for cessation of AGE linked vomiting, the possible reasons could be that when Ondansetron is administered orally, it is absorbed in the GI tract^14^ and as it is a “serotonin 5-HT3 receptro antagonist, it suppresses the vomiting centers in the brain and blocks afferent depolarization of vagal nerves peripherally in the intestine^15^,^16^ that might be provoking emesis responses among patients of AGE.^17^ As Ondansetron is believed to reduce emesis, it might be directly improving or the oral intake of ORT which further goes on to reduce the need for IV rehydration as well as hospitalization.^18^-^20^

The strengths of this study include, being the 1^st^ study from a resource limited setting of Pakistan comparing efficacy of Domperidone versus Ondansetron involving children aged up to 12 years. A uniform dosing and treatment protocol was used in both study groups so chances of bias were also reduced. As this study was conducted over a period of one year, so we had included children from different seasons and weather.

### Limitations of the study

Open label design was one of the limitations. As this was a single center study, further double blind randomized controlled trial is required to further establish the efficacy of Ondansetron versus Domperidone among children with AGE having vomiting.

## CONCLUSION

In comparison to Domperidone, Ondansetron was found to have better efficacy aiming cessation of AGE associated vomiting among children with mild to moderate dehydration.

### Authors Contribution:

**TA:** Conceived, Data Collection, Responsible for data’s integrity and authenticity.

**UZ:** Data Analysis, Drafting.

**BS:** Literature Review, Discussion.
